# A Non-invasive Method to Diagnose Lung Adenocarcinoma

**DOI:** 10.3389/fonc.2020.00602

**Published:** 2020-04-29

**Authors:** Mengmeng Yan, Weidong Wang

**Affiliations:** ^1^Urban Vocational College of Sichuan, Chengdu, China; ^2^School of Medicine, University of Electronic Science and Technology of China, Chengdu, China; ^3^Department of Radiation Oncology, Sichuan Cancer Hospital and Institute, Chengdu, China; ^4^Radiation Oncology Key Laboratory of Sichuan Province, Chengdu, China

**Keywords:** radiomics, texture analysis, lung adenocarcinoma, multi-instance learning, lung cancer histological types

## Abstract

**Purpose:** To find out the CT radiomics features of differentiating lung adenocarcinoma from another lung cancer histological type.

**Methods:** This was a historical cohort study, three independent lung cancer cohorts included. One cohort was used to evaluate the stability of radiomics features, one cohort was used to feature selection, and the last was used to construct and evaluate classification models. The research is divided into four steps: region of interest segmentation, feature extraction, feature selection, and model building and validation. The feature selection methods included the intraclass correlation coefficient, ReliefF coefficient, and Partition-Membership filter. The performance metrics of the classification model included accuracy (Acc), precision (Pre), area under curve (AUC), and kappa statistics.

**Results:** The 10 features (First order shape features: Sphericity and Compacity, Gray-Level Run Length Matrix: Short-Run Emphasis, Low Gray-level Run Emphasis, and High Gray-level Run Emphasis, Gray Level Co-occurrence Matrix: Homogeneity, Energy, Contrast, Correlation, and Dissimilarity) showed the most stable and classification capability. The 6 classifiers, Logistic regression classifier (LR), Sequence Minimum Optimization algorithm, Random Forest, KStar, Naive Bayes and Random Committee, have great performance both on the train and the test sets, and especially LR has the best performance on the test set (Acc = 98.72, Pre = 0.988, AUC = 1, and kappa = 0.974).

**Conclusion:** Lung adenocarcinoma can be identified based on CT radiomics features. We can diagnose lung adenocarcinoma with CT non-invasively.

## Introduction

Medical imaging can assess the characteristics of human tissues non-invasively and is often used in the diagnosis, treatment guidance and monitoring of tumors in clinical practice. And radiomics can extract and quantify the differences in tumor tissues (1–4).

**Figure 1:**
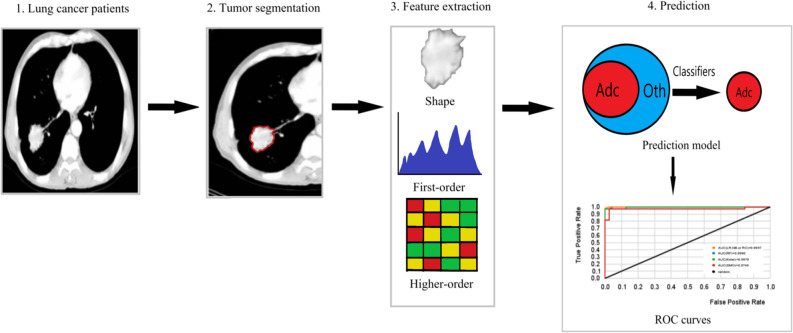
The pipeline of our proposed radiomics analysis. (1) Original images of lung cancer patients. (2) Tumor area of interest (ROI) segmentation of each slice of CT. (3) Extraction of shape, first-order features and higher-order features from the ROI. (4) Prediction model building based on machine learning classifiers, ROC curves used to assess the model performance. Adc is lung Adenocarcinoma, and Oth are other lung cancer histological subtypes.

The radiomics workflow is usually divided into four steps (1, 5, 6): The first step is image collection and segmentation. All kinds of medical image formats are supported by radiomics, but in terms of the number of studies, CT radiomics has the largest number of studies, followed by PET, MR, and ultrasound. The segmentation methods include manual segmentation and semi-automatic segmentation. The second step is feature extraction. This part of the work is easy to standardize. And the third step is feature selection. Feature selection methods are divided into supervised learning and unsupervised learning. No matter which type of feature selection, stability evaluation and performance evaluation should be carried out. The influence of feature redundancy varies with the algorithms. The final step is model building. The algorithms of model building can be roughly divided into machine learning and deep learning, and the selection index is data quantity. Besides, basic medical statistical methods, such as hypothesis testing, can also be used for radiomics analysis. Figure 1 shows the pipeline of our proposed radiomics analysis.

The histological type diagnosis of lung cancer is fundamental in guiding patient management. Lung biopsy is a well-established method for the differential diagnosis of lung lesions (7), but it is expensive and invasive. Lung Adenocarcinoma (Adc) is the most common subtype of lung cancer (8), and diagnosing Adc by biopsy is not beneficial to the patients unfit for the invasive diagnostic procedure. So it is important to diagnose Adc from others (binary classification) by radiomics so that the patients will get accurate treatment earlier without invasive. In addition, it could be the basis to develop a multiple class classification model to reduce or avoid the use of invasive diagnostic methods.

This paper tests the hypothesis that Adc can be predicted from another lung cancer histological type (Oth) by radiomics. To invest the evidence of that, we analyzed three independent lung cancer cohorts, built some lung Adc classifiers that can differentiate Adc from Oth without considering the clinical parameters. To our knowledge, this work is the first radiomics-based study to predict Adc from Oth (including squamous cell carcinoma, other primary lung cancer and metastases), and the proposed models are non-invasive and cost-effectiveness.

## Result

### The Most Stable Features With High Classification Capability

Table 1.1 lists the 30 most stable features ranked by intraclass correlation coefficient (the threshold value is 0.85, *p* < 0.01) in RIDER (9) data set. Most of the extracted radiomics features have good stability. Based on the 30 most stable radiomics features, the ReleifF (KenjiKira et al. presented at the 1992 Machine Learning Proceedings) algorithm (10 times cross-validation) shows 10 features with classification ability (threshold value is 0.01) in Table 1.2. The features based on shape, Gray Level Co-occurrence Matrix (GLCM), and Gray-Level Run Length Matrix (GLRLM) had better classification ability, where Sphericity and Compacity based on shape describe the tumor shape such as spherical, round or elongated, Contrast_GLCM describes the local differences and higher value stands for greater difference between neighboring voxels, SRE_GLRLM is a measure of short run length distribution, and larger values represent better texture structure.

**Table 1:** The analysis results of three independent data sets.

**Class**	**Features**
**1.1 The 30 most stable features on RIDER data set**	
^a^FH	Skewness, kurtosis, energy
^b^FS	Sphericity, compacity, volume
^c^GLZLM	Short-zone emphasis, high gray-level zone emphasis, short-zone low gray-level emphasis, short-zone high gray-level emphasis, long-zone low gray-level emphasis, zone length non-uniformity, low gray-level run emphasis, high gray-level run emphasis
^d^GLRLM	Short-run emphasis, long-run emphasis, low gray-level run emphasis, high gray-level run emphasis, short-run high gray-level emphasis
^e^NGLDM	Coarseness, contrast
^f^GLCM	Homogeneity, energy, contrast, correlation, dissimilarity
Conventional Indices	minValue, maxValue, meanValue, stdValue
**1.2 The 10 most stable features with classification capability on Lung 1 data set**	
^b^FS	Sphericity, compacity
^d^GLRLM	Short-run emphasis, low gray-level run emphasis, high gray-level run emphasis
^f^GLCM	Homogeneity, energy, contrast, correlation, dissimilarity
**Classifiers**	**Accuracy(%)**
**1.3 Accuracy ratio of 6 machine learning classifiers on Lung 2 test set**	
^g^LR	**98.72**
^h^RC	**98.72**
^i^SMO	97.44
^j^RF	97.44
^k^NB	**98.72**
Ksrar	96.15

a *First-order features-histogram*.

b *First order features-shape*.

c *Gray-Level Zone Length Matrix, provides information on the size of homogeneous zones for each gray-level in 3 dimensions*.

d *Gray-Level Run Length Matrix, gives the size of homogeneous runs for each gray level. This matrix is computed for the 13 different directions in 3D (4 in 2D) and each of the 11 texture indices derived from this matrix, the 3D value is the average over the 13 directions in 3D (4 in 2D)*.

e *Neighborhood Gray-Level Different Matrix, corresponds to the difference of gray-level between one voxel and its 26 neighbors in 3 dimensions (8 in 2D)*.

f *Gray Level Co-occurrence Matrix, takes into account the arrangements of pairs of voxels to calculate textural indices*.

g *logistic regression*.

h *Random Committee*.

i *Sequential minimal optimization*.

j *Random Forest*.

k *Naive Bayes*.

*The best accuracy ratios are highlighted in bold*.

Partition-Membership filter (PMF) used the random Committee algorithm as the partition generator to divide the 10 features into 1940 partitions (Supplementary Material). The minimum feature subset contained 122 partitions with the highest classification capability selected by correlation-based feature subset selection (CFS).

### Model Performance

Table 1.3 shows the accuracy ratios in 6 machine learning classifiers on the test set, including Logistic regression classifier (LR), Sequence Minimum Optimization algorithm (SMO), Random Forest (SF), KStar, Naive Bayes (NB) and Random Committee (RC). All of them have a great performance on the test set, and especially LR, RF, and NB get the highest accuracy of 98.72%. It also stands for the great classification capability of those 10 features in diagnosing Adc.

**Figure 2:**
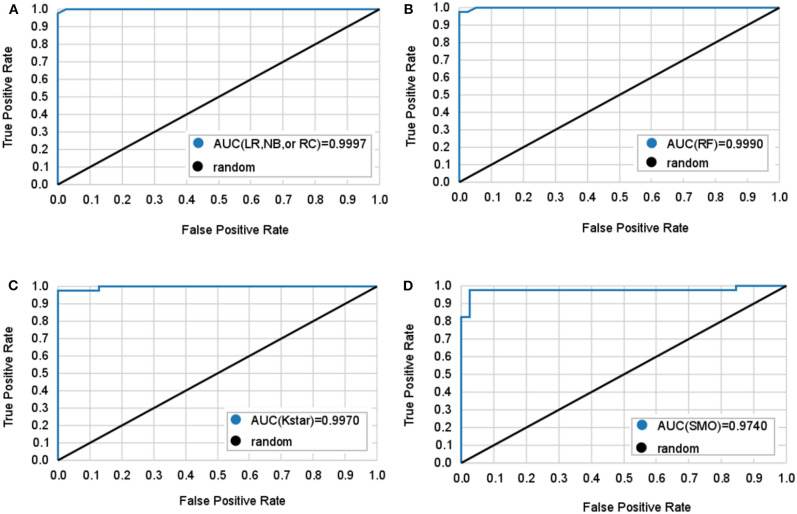
Mean ROC curves obtained by six machine learning models for predicting lung adenocarcinoma. The black diagonal line in the diagram is the random line which is the worst possible performance a model can achieve. **(A)** Logistic regression (LR), naive bayes (NB), and random committee (RC) classifiers all have the same AUC. **(B)** Random forest (RF) classifier. **(C)** Kstar classifier. **(D)** Sequential minimal optimization (SMO) classifier.

Table 2 and Figure 2 show 6 classifiers with great performance on the train and the test sets. The best performance metrics for each set are highlighted in bold. As a whole, the 6 classifiers have excellent classification performance both on the train and the test sets, which shows that they can not only diagnose Adc but also rule out Oth with high accuracy. There is no significance between prediction models (*P* > 0.05), which can be inferred that the selected 10 features have great ability to diagnose Adc. On the test set, the Kappa statistics are approximately equal to 1 for all models shows that the models have great stability, and the minimum value is 0.923 (Kstar). Meanwhile, the mean absolute errors (MAE) are approximately equal to 0, and the maximum value is 0.09 (Kstar).

**Table 2:** Performance metrics of 6 classifiers on the train set and test set.

**Performance**	**Accuracy (%)**	**^l^TPR**	**^m^TNR**	**Precision**	**^n^AUC**	**Kappa**	**^°^MAE**
^g^LR							
Train set	**98.70**	**0.980**	0.993	**0.987**	0.996	**0.973**	**0.02**
Test set	**98.72**	**0.987**	**1.000**	**0.988**	**1.000**	0.974	**0.01**
^h^RC							
Train set	96.40	0.967	0.961	0.964	**0.997**	0.928	0.07
Test set	**98.72**	0.974	**1.000**	**0.988**	**1.000**	**1.000**	0.05
^i^SMO							
Train set	97.72	0.961	0.993	0.978	0.977	0.954	0.02
Test set	97.44	0.974	0.974	0.974	0.974	0.949	0.03
^j^RF							
Train set	97.72	0.974	0.980	0.977	**0.997**	0.954	0.10
Test set	97.44	0.974	0.974	0.974	0.999	0.949	0.08
^k^NB							
Train set	97.01	0.948	0.993	0.972	0.994	0.942	0.06
Test set	**98.72**	0.974	**1.000**	**0.988**	**1.000**	0.974	0.05
Kstar							
Train set	96.08	0.922	**1.000**	0.964	0.997	0.921	0.10
Test set	96.15	0.949	0.949	0.974	0.997	0.923	0.10

g *logistic regression*.

h *Random Committee*.

i *Sequential minimal optimization*.

j *Random Forest*.

k *Naive Bayes*.

l *True Positive Rate*.

m *True Negative Rate*.

n *Area under curve*.

° *Mean absolute error*.

*The best performance metrics for each set are highlighted in bold*.

LR classifier has the best performance on the test set, it also has the highest accuracy, true positive rate (TPR), true negative rate (TNR), precision, and lowest MAE on train set. Followed by RC and NB, which have the highest TNR, precision, and area under curve (AUC) on the test set. It is important to diagnose Adc from Adcs so that patients will get accurate treatment earlier. Table 2 shows LR has great ability to diagnose Adc from Adcs with over 98% accuracy on the test set. And LR, RC, and NB have perfect accuracy in diagnosing Oth from Oths.

## Discussion

Radiomics provides a non-invasive and fast method to predict clinical outcomes. It could not only support precision medicine but also be a household diagnostic tool. It is an effective way to use radiomics to support therapy decision-making, which will advance personalized medicine. Radiomics has been applied to a variety of organs and systems such as brain, breast, lung, heart, liver, kidney, adrenal gland, cervix, limbs, and prostate (6, 10, 11). For example, Chaddad et al. (6, 12) proposed a multiscale texture features to predict progression free and overall survival in patients newly diagnosed with glioblastoma, they also reviewed the clinical implementation of radiomic in the current management of glioblastoma, which is important for advancing the personalized treatment of glioblastoma patients.

It has been proved the correlation between radiomics features and tumor phenotype (12–22). Many studies have found Adc can be predicted by radiomics (22–28). Tang et al. (27) developed a radiomics model to discriminate Adc from squamous cell carcinoma (Sqc) with an AUC of 0.82, Yang et al. (24) developed an LR model to predict lymph node metastasis in solid Adc with an AUC of 0.86. Remeo et al. (23) studied ground-glass nodules diagnosis by radiomics, and found radiomics classifier may be a reliable tool for clinical decision. Ferreira-Junior et al. (28) found some radiomics features associated with Adc and squamous cell carcinoma, and got an AUC of 0.88 with a machine learning model.

However, from the data set point of view, the data sets of these studies only contain Adc and Sqc, and in clinical we can't rule out the existence of other subtypes before lung biopsy. So from the perspective of clinical diagnosis, the study of predicting Adc should include all subtypes of lung cancer as many as possible. Besides, among these studies, the performance of CT radiomics models still needs to be improved.

The proposed radiomics models showed great performance in diagnosing Adc both on the train and the test sets. The models are available and can be applied in Weka.

In this study, lung cancer patients with various histological subtypes were included in the patient cohorts. We used stratified random sampling to balance the covariates. In feature selection, we first test the stability of the feature using the public RIDER data set. Then pick up the features with classification capability. The selected 10 features show excellent classification ability after PMF and CFS. PMF was used for transforming features and CFS is good at picking the most representative minimum feature subset. It has been proved that PMF can not only solve the problem of binary classification but also improve the accuracy of classification (29, 30). Meanwhile, in order to avoid over-fitting as much as possible, the train and the test sets were divided with stratified random sampling to keep them balanced. For model development, independent data sets were used for feature selection and model construction, and cross validation method was used for resampling. In model selection, we used many classifiers to show the classification ability of selected features, including three frequently used classifiers LR, RF, and NB. RF contains multiple trees, even if some trees have over-fitting, it can reduce over-fitting by voting or averaging. Many radiomics studies used RF for classification. RC is an ensemble method, it will build an ensemble of randomizable base classifiers. Each base classifier is built using a different random number seed. The final prediction is a straight average of the predictions generated by the individual base classifiers. Kstar is an instance-based learner using an entropic distance measure to solve the smoothness problem. SMO is used for training a support vector classifier, which has good robustness and generalization ability.

A few issues regarding the stability and reproducibility of the radiomics features have been raised in recent years (31–33). Multiple parameter changes (e.g., slice thickness) in general produce greater measurement errors. Therefore, some parameters such as slice thickness, dose, kernel, and segmentation methods should not be altered to assess the features of a radiomics model. In this case, we selected the most stable features across test-retest. To find the most representative feature subset and reduce the running time of the classifiers, we used CFS to pick the most representative minimum feature subset. CFS uses heuristic and best-first search methods to evaluate feature subsets and filters out features that are highly correlated with classes but have the lowest correlation with each other.

Although we try our best to reduce random errors and ensure the correctness of statistical analysis in this study, there are several limitations. Two cohorts in our study are from public data sets, so we cannot accurately estimate the size and direction of systematic bias. The area of interest of the Lung 1 data set and the Lung 2 data set are delineated in different ways, which will lead to measurement errors. Besides, we need more cases to improve the classification model.

In conclusion, CT based radiomics can identify Adc. Therefore, we can distinguish Adc only from CT images. We will include multicenter data to improve the classifier and make it a clinical diagnostic tool.

## Materials and Methods

Our work was approved by the institutional Ethics Committee.

The tools used for statistical analysis were IBM SPSS Statistics 25.0 (USA), and Weka (Frank et al. presented at the 2009 Data mining and knowledge discovery handbook) (Weka v3.8.3, Hamilton, New Zealand).

### Data Sets

We analyzed three independent data sets including a public RIDER data set (9), a lung cancer cohort from our institute (Lung 1), and a public radiomics features data set (Lung 2) (4), Table 3 shows Patient characteristics of Lung 1 and Lung 2. Patients characteristics in detail, criteria for patient selection, and CT scan protocol of Lung 2 have been already published (4).

**Table 3:** Patient characteristics.

**Characteristics**	**Lung 1**	**Lung 2**
Size, N	180	535
Mean Age	66	69
Gender (%)		
Female	30.6	33.3
Male	69.4	66.7
Histological type, N		
Adenocarcinoma	90	193
Squamous cell carcinoma	30	132
Other primary lung cancer	30	79
Metastases	30	131
^a^The significance of radiomics features, N		
*P* ≤ 0.05	^b^8	
*P*> 0.05	33	

a *Paired t-test with 95% Confidence Interval, two-tailed*.

b *They are Volume_Shape, Long-Run Emphasis_Gray-Level Run Length Matrix, Coarseness_Neighborhood Gray-Level Different Matrix, Contrast_Neighborhood Gray-Level Different Matrix, Long-Zone Low Gray-level Emphasis_Gray-Level Zone Length Matrix, Zone Length Non-Uniformity_Gray-Level Zone Length Matrix, Low Gray-level Run Emphasis_Gray-Level Zone Length Matrix, High Gray-level Run Emphasis_Gray-Level Zone Length Matrix*.

The RIDER data set consists of 31 non-small cell lung cancer patients with two CT scans obtained in an interval of about 15 min. We use this data set to evaluate the stability of features for test-retest.

Lung 1 data set consists of 180 lung cancer patients (adenocarcinoma: squamous cell carcinoma: other types of lung cancer: metastasis = 3:1:1:1) from our institutional database in 2010–2018. For these patients, CT images, manual delineations, and clinical data were available. The criteria for patient selection are the same as Lung 2. We use this data set for feature selection.

Lung 2 data set consists of 535 lung cancer patients. For these patients, texture features were available. We used this data set for model building and validation. In order to keep the data class balanced on the train and the test sets(adenocarcinoma: squamous cell carcinoma: other types of lung cancer: metastasis = 3:1:1:1) and include as many patients as possible, we randomly divided it into train set (*n* = 306) and test set (*n* = 78). Specific patients were selected by pseudorandom numbers.

According to the lung histological diagnosis, the data class was divided into Adc and Oth (including squamous cell carcinoma, other primary histological subtypes, and metastatic lung cancer). The research of the data set can be divided into two stages: training phase and validation phases. The training phase included CT image acquisition, texture feature extraction, feature selection, and model building. The validation phase included model testing and performance evaluation.

### CT Image Acquisition and Texture Feature Extraction

The acquisition and processing of Lung 1 and Lung 2 CT images were carried out following Image Biomarker Standardization Initiative (IBSI) (34). The volume of interest (VOI) of the lung 1 data set is made by two experienced radiologists independently. Before the work, the physiologists did not know the histological subtype (blindness) of the target patient. For the inconsistent segments, they will be segmented again after comparison until the outcomes are consistent. The VOI of the Lung 2 data set is segmented (semi)automatically.

LIFEx package (35) used to extract texture features. It can efficiently perform textural analysis and radiomics feature measurements from CT images. 41 features were extracted from CT images.

### Feature Selection

The stability of the radiomics features was evaluated by using the RIDER data set. For each patient, we extracted image features from two scans. The stability of each feature was calculated using the intraclass correlation coefficient, where the higher the intraclass correlation coefficient corresponds to the more stable feature (1).

Based on the results of feature stability, The ReliefF algorithm (ReliefF Attribute Eval with Ranker in WEKA) was used to remove the irrelevant features from the lung 1 data set.

The selected features were filtered by propositionalization and partition using the Partition-Membership filter (Partition Membership Filter with option Random Committee in Weka) on Lung 2 train and test sets. It can apply any partition generator to a given feature vector to get these filtered vectors for all instances, and the filtered instances are composed of these values plus class attribute and make as sparse instances (29).

Then we used CFS to filter the results. The CFS can select the minimum feature set that is highly related to the classes. In this feature set, there is a low correlation between features, so feature redundancy can be reduced. That is to say, the final result is the feature set with the highest prediction ability, and there is a low correlation between the features in this feature set.

### Model Building and Performance Evaluation

We used 6 machine learning classifiers, including LR(logistic with options -R 1.0E-8 -M−1 in Weka), ensemble learning classifier RF (Random Forest with options -K 0 -M 1.0 -V 0.001 -S 1 in Weka), Sequential minimal optimization(SMO with options -C 1.0 -L 0.001 -P 1.0E-12 -N 1 -V−1 -W 1 -K in Weka), NB (naïve Bayes in Weka), RC (Random Committee with options -S 1 -num-slots 1 -I 10 -W in Weka), and KStar (Kstar in Weka) with 10-folds cross validation. The performance metrics of the classification model included TPR, TNR, accuracy, precision, AUC, kappa statistics, and MAE. Table 4 shows the calculation formulas of these metrics.

**Table 4:** The calculation formulas of performance metrics.

**Metric**	^*^Formula
TPR	$\frac{TP}{TP+FN}$
TNR	$\frac{\text{TN}}{\text{TN}+\text{FP}}$
Accuracy	$\frac{\text{TP}+\text{TN}}{\text{TP}+\text{FP}+\text{TN}+\text{FN}}$
Precision	$\frac{\text{TP}}{\text{TP}+\text{FP}}$
AUC	$\int_{\text{x}=\text{0}}^{\text{1}}TPR\left(FPR^{-1}\left(x\right)\right)dx$, where x_1_ is the score for a positive instance and x_0_ is the score for a negative instance.
Kappa	$\text{Kappa}=\frac{{\text{P}}_{o}-P_{e}}{1-P_{e}}$, ${\text{P}}_{e}=\frac{\text{P}\left(TP+FP\right)+N\left(TN+FN\right)}{{\left(T+N\right)}^{2}}$ where P_o_ = Accuracy,
MAE	$\frac{\text{1}}{n}\overset{n}{\underset{i=1}{\sum }}|p\left(i\right)-a\left(i\right)|$, where p(i) is the prediction case, and a(i) is real case, n is the total cases.

* *TP is true positive, it means that the outcome from a prediction is lung adenocarcinoma (Adc) and the actual value is also Adc. FN is false negative, it means that the prediction outcome is another lung cancer histological type(Oth) while the actual value is Adc. TN is true negative, it means that both the prediction outcome and the actual value are Oth. FP is false positive, it means that the outcome from a prediction is Adc while the actual value is Oth. P is condition positive, N is condition negative, and MAE is the mean absolute errors. TPR is true positive rate, it measures the proportion of actual patients with Adc that are correctly identified. A negative result in a test with high TPR is useful for ruling in disease, it signifies a high probability of the presence of Oth. TNR is true negative rate, it measures the proportion of actual patients with Oth that are correctly identified. A test with 100% TNR will recognize all patients with Oth by testing negative, and a positive test result would definitively rule out the presence of Oth in a patient*.

## Data Availability Statement

All datasets generated for this study are included in the article/Supplementary Material.

## Author Contributions

All authors listed have made a substantial, direct and intellectual contribution to the work, and approved it for publication.

## Conflict of Interest

The authors declare that the research was conducted in the absence of any commercial or financial relationships that could be construed as a potential conflict of interest.
