# Corrigendum: A Non-invasive Method to Diagnose Lung Adenocarcinoma

**DOI:** 10.3389/fonc.2020.01515

**Published:** 2020-08-20

**Authors:** Mengmeng Yan, Weidong Wang

**Affiliations:** ^1^Urban Vocational College of Sichuan, Chengdu, China; ^2^School of Medicine, University of Electronic Science and Technology of China, Chengdu, China; ^3^Department of Radiation Oncology, Sichuan Cancer Hospital and Institute, Chengdu, China; ^4^Radiation Oncology Key Laboratory of Sichuan Province, Chengdu, China

**Keywords:** radiomics, texture analysis, lung adenocarcinoma, multi-instance learning, lung cancer histological types

In the published article, there was an error regarding the affiliations for Mengmeng Yan. As well as having affiliation 2, School of Medicine, University of Electronic Science and Technology of China, Chengdu, China, they should also have Urban Vocational College of Sichuan, Chengdu, China.

The authors apologize for this error and state that this does not change the scientific conclusions of the article in any way. The original article has been updated.

